# Comparative In Vitro Evaluation and Osteogenic Mechanisms of Representative Bone Graft Substitutes: Bioactive Glass, Beta-Tricalcium Phosphate, and Deproteinized Bovine Bone

**DOI:** 10.3390/jfb17070312

**Published:** 2026-06-26

**Authors:** Jianhang Yuan, Zimeng Li, Ziwei Dai, Yingyue Chai, Zixuan You, Shang Xie, Yifan Kang, Xiaofeng Shan, Zhigang Cai

**Affiliations:** 1 Department of Oral and Maxillofacial Surgery, Peking University School and Hospital of Stomatology, Beijing 100081, China; 2411210553@pku.edu.cn (J.Y.); lizimeng1992@pku.edu.cn (Z.L.);; 2 National Center for Stomatology, Beijing 100081, China; 3 National Clinical Research Center for Oral Diseases, Beijing 100081, China

**Keywords:** bioactive glass, bone substitutes, osteogenesis, tissue engineering, transcriptome

## Abstract

**Objectives:** Autologous bone grafting remains the gold standard for maxillofacial reconstruction but is limited by tissue scarcity and donor-site morbidity. Consequently, substitutes like bioactive glass (BG), beta-tricalcium phosphate (β-TCP), and deproteinized bovine bone (DBB) are widely used. However, comprehensive mechanistic comparisons among them remain scarce. **Materials and Methods:** We systematically evaluated these substitutes under standardized in vitro conditions to compare their physicochemical transformations, degradation profiles, biological performances, and underlying osteogenic molecular pathways. **Results:** In simulated body fluid, BG underwent rapid hydroxyapatite mineralization, whereas the highly porous DBB and dense β-TCP remained structurally inert. Degradation assays revealed BG exhibited the fastest mass loss and ion release, β-TCP showed intermediate degradation, and DBB maintained high in vitro structural stability. Biologically, all materials showed favorable cytocompatibility and comparable angiogenic potential. However, BG demonstrated significant antibacterial activity (*E. coli*, *S. aureus*) and a strong potential to enhance osteogenic differentiation, significantly upregulating the protein-level expression of RUNX2 and OCN, alongside the transcriptional upregulation of *Bmp2*, *Runx2*, and *Ocn*. Transcriptomic profiling and pharmacological validation suggest that the enhanced osteogenic performance of BG might be associated with specific regulatory pathways, supporting the hypothesis that the suppression of NF-κB-mediated inflammation and the activation of the ECM-Integrin-FAK mechanotransduction axis play potential roles. **Conclusions:** BG offers high bioactivity and notable potential to enhance osteogenic differentiation in vitro but degrades rapidly. DBB ensures structural durability without intrinsic osteoinductivity, and β-TCP provides a balanced, intermediate profile. These in vitro mechanistic insights provide a theoretical foundation for future in vivo evaluations and designing next-generation bone scaffolds.

## 1. Introduction

Jawbone defects, which frequently arise from alveolar bone resorption, periodontitis, trauma, and tumor resection, remain a formidable challenge in oral implantology and maxillofacial surgery [1,2]. While autologous bone grafting is considered the gold standard due to its excellent bone regeneration results, its widespread clinical application is constrained by donor-site morbidity and limited tissue availability [2,3]. Consequently, a variety of artificial bone substitutes have been developed. Ideally, these substitutes should not only occupy the defect space but also actively modulate the local microenvironment to promote osteogenesis and prevent infection [4]. Among the diverse array of available options, bioactive glass (BG), β-tricalcium phosphate (β-TCP), and deproteinized bovine bone (DBB) have widely emerged as the classical representatives of silicate-based synthetic materials, phosphate-based ceramics, and natural xenografts, respectively [1,3,5].

Owing to their distinct compositions and manufacturing processes, these three classes of materials exhibit fundamentally divergent biological behaviors and clinical outcomes. While BG-based materials are renowned for their exceptional ion-driven bioactivity [6], they often face clinical challenges regarding rapid degradation and long-term volumetric stability [7,8]. Conversely, alternatives such as β-TCP [9,10,11] and naturally derived DBB [12,13,14] offer different balances of structural preservation, osteoconductivity, and physiological resorption kinetics.

Despite the widespread clinical application of these materials, comprehensive, head-to-head comparisons evaluating them within a unified experimental framework remain surprisingly scarce. The existing literature predominantly focuses on single-material characterizations or binary comparisons, resulting in a fragmented understanding of their relative physicochemical and biological hierarchies [15,16,17,18]. Furthermore, although phenotypic variations such as distinct mineralization capacities are widely recognized, the precise molecular mechanisms orchestrating these divergent cellular responses remain largely unexplored. Specifically, the comprehensive transcriptomic landscapes and specific signaling cascades modulated by these distinct material classes have yet to be fully elucidated.

To bridge this critical gap, the present research establishes a standardized experimental framework to systematically investigate three representative commercial bone graft powders under strictly identical in vitro conditions: Perioglas^®^ (BG), Bio-Lu^®^ (β-TCP), and Bio-Oss^®^ (DBB). In this study, a comprehensive array of quantitative and qualitative data was collected, encompassing physicochemical transformations, dynamic in vitro degradation, hemocompatibility, angiogenic potential, and antibacterial efficacy. Furthermore, we employed high-throughput RNA sequencing (RNA-seq) to unravel the specific transcriptomic profiles and signaling pathway modulations in mouse bone marrow mesenchymal stem cells (mBMSCs). Ultimately, this study aims to provide a rigorous in vitro comparative screening to elucidate how material composition and structure initially direct cell fate. While these simplified in vitro models cannot fully replicate the physiological complexity of in vivo bone healing, thereby serving as a valuable theoretical reference for clinical material selection and guiding the rational design of next-generation bioactive scaffolds [7,8,10,19,20,21,22].

## 2. Experimental Methods

### 2.1. Preparation of Materials

To closely mimic clinical usage scenarios, all commercial bone graft substitutes were utilized as supplied by the manufacturers without further modification. The materials included: Perioglas^®^ (NovaBone Products, LLC, Alachua, FL, USA; hereinafter referred to as BG; granule size: 90–710 µm), Bio-Lu^®^ (Bio-Lu Biomaterials Co., Ltd., Shanghai, China; hereinafter referred to as β-TCP; granule size: <1 mm), and Bio-Oss^®^ (Geistlich Pharma AG, Wolhusen, Switzerland; hereinafter referred to as DBB; granule size: 0.25–1 mm). All material quantities were precisely weighed using an analytical balance (OHAUS, CP64C).

### 2.2. BET Surface Area and Pore Size Analysis

The specific surface area and pore size distribution of the as-received powders were characterized using a surface area analyzer (ASAP 2460, Micromeritics, Norcross, GA, USA) through nitrogen (N_2_) adsorption–desorption isotherms measured at −196 °C. The specific surface area was calculated utilizing the Brunauer–Emmett–Teller (BET) method, and the pore size distribution was derived from the desorption branch using the Barrett–Joyner–Halenda (BJH) model.

### 2.3. Simulated Body Fluid (SBF) Immersion Experiment

To assess microstructural changes under simulated physiological conditions, the powder samples were immersed in simulated body fluid (SBF; pH 7.4, G0390, Solarbio, Beijing, China). To mimic dynamic physiological fluid turnover and prevent localized ion saturation, the supernatant was completely replaced with fresh SBF on day 7. The suspensions were incubated at 37 °C under continuous orbital shaking (100 rpm). At predetermined time points (days 1, 3, 7, and 14), the residual solid powders were collected, thoroughly rinsed with deionized water, and dried to a constant mass prior to detailed microstructural characterization. To ensure statistical reliability, all immersion experiments at each time point were performed in independent triplicates (*n* = 3).

Scanning Electron Microscopy (SEM) Analysis: The surface morphology of both as-received and SBF-soaked powders was examined using field-emission scanning electron microscopy (FE-SEM; SU8010, Hitachi, Tokyo, Japan). Powders were mounted onto aluminum stubs using conductive carbon tape. Ten randomly selected representative fields were captured per sample.

X-ray Diffraction (XRD) Analysis: The crystalline phases of the as-received and SBF-soaked powders were analyzed using an X-ray diffractometer (Miniflex600, Rigaku, Tokyo, Japan). Samples were scanned continuously from 10° to 80° (2θ) with a step size of 0.02° at a scanning rate of 2°/min. The resulting diffraction patterns were processed using Jade 9.0 software (Materials Data, Inc., Livermore, CA, USA) and cross-referenced against the International Center for Diffraction Data (ICDD) PDF database to identify phase compositions.

### 2.4. In Vitro Degradation Assay

To evaluate the degradation kinetics, powder samples were immersed in sterile Tris-HCl buffer (50 mM, pH 7.4, Beyotime Biotech, Shanghai, China) at a specific solid-to-liquid ratio in strict accordance with the ISO 10993-12 standard [23]. To mimic dynamic physiological fluid turnover and prevent localized ion saturation, the entire supernatant was completely replaced with fresh Tris-HCl buffer on day 7. The suspensions were incubated at 37 °C under continuous orbital shaking (100 rpm). At predetermined time points (days 1, 3, 7, and 14), the solid phase was separated via centrifugation. The resulting supernatants were collected for subsequent analyses, while the residual solid powders were thoroughly rinsed with deionized water and dried to a constant mass. To ensure statistical reliability, all degradation experiments at each time point were performed in independent triplicates (*n* = 3).

pH Measurement: The pH variation in the collected supernatants at each specific time point was accurately recorded utilizing an ultra-micro pH meter (Mettler Toledo, Greifensee, Switzerland).

Weight Loss Quantification: The residual solid powders collected after centrifugation were thoroughly rinsed with deionized water to remove residual buffer salts, dried to a constant mass, and precisely weighed to calculate the structural mass loss percentage.

Ion Release Kinetics (ICP-OES): The concentrations of released calcium (Ca), silicon (Si), and phosphorus (P) ions in the supernatants were quantitatively determined via inductively coupled plasma optical emission spectrometry (ICP-OES; 5800, Agilent, Santa Clara, CA, USA).

### 2.5. In Vitro Coagulation Assay

Hemocompatibility was quantitatively and macroscopically evaluated using EDTA-anticoagulated rabbit whole blood (SBJ-AC-RAB02, Sbjbio Life Sciences, Nanjing, Jiangsu, China). To initiate the coagulation cascade for all subsequent assays, the blood was recalcified and activated with an aqueous CaCl_2_ solution (0.1 mol/L; Macklin, Shanghai, China). To ensure statistical reliability, all coagulation experiments were performed in independent triplicates (*n* = 3).

Blood Clotting Index (BCI) Test: The BCI was utilized to quantify the extent of thrombus formation. The materials were co-incubated with the recalcified blood at 37 °C for predefined intervals (5, 15, 30, and 60 min). The coagulation process was subsequently arrested, and uncoagulated red blood cells were lysed by adding deionized water to each sample. The absorbance of the resulting free hemoglobin solution was recorded at 540 nm using a microplate reader (MK3, Thermo Fisher Scientific, Waltham, MA, USA). The BCI was calculated using the following equation:

$$BCI(\%)=\frac{A_{sample}}{A_{blood}}\;\times 100\%$$

where *A*_sample_ represents the absorbance of the supernatant from the material-treated group, and *A*_blood_ represents the absorbance of the reference control.

Clotting Time Test: To temporally determine the macroscopic clotting time, each material was similarly co-incubated with the recalcified whole blood at 37 °C. At predefined intervals, the samples were gently rinsed with phosphate-buffered saline (PBS) to remove uncoagulated blood, and the time required for stable clot formation on the material surface was precisely recorded.

### 2.6. Cell Collection and Subculture

**Figure 1:**
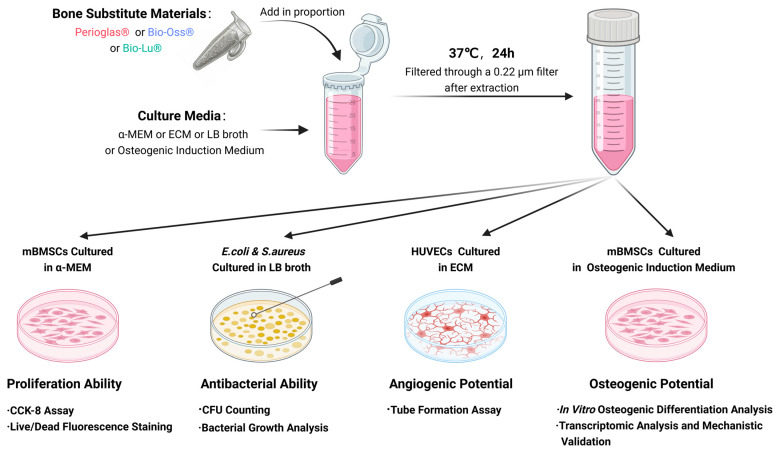
**Schematic illustration of the in vitro experimental design.** The workflow details the standardized preparation of material extraction solutions and their subsequent application in downstream biological evaluations. These systematic assays assess cellular proliferation and cytocompatibility (mBMSCs), antibacterial efficacy (*E. coli* and *S. aureus*), baseline angiogenic potential (HUVECs), and osteogenic differentiation coupled with transcriptomic mechanistic explorations.

Primary mouse bone marrow mesenchymal stem cells (mBMSCs; passages 3–5, CP-M131) and human umbilical vein endothelial cells (HUVECs; passages 3–5, CP-H082) were purchased from Procell Life Science & Technology Co., Ltd. (Wuhan, China). The mBMSCs were cultured in α-Minimum Essential Medium (α-MEM, Gibco, Grand Island, NY, USA), while the HUVECs were maintained in specific Endothelial Cell Medium (ECM, Gibco) containing endothelial cell growth supplement (ECGS). Both culture media were supplemented with 10% fetal bovine serum (FBS, Gibco) and a 1% penicillin–streptomycin mixture (ST488S, Beyotime). All cells were routinely expanded at 37 °C in a humidified incubator with a 5% CO_2_ atmosphere. For the subsequent biological evaluations, both mBMSCs and HUVECs were treated with the respective material extraction solutions prepared in strict accordance with the ISO 10993-12 standard guidelines. Specifically, considering the irregularly shaped and slowly degrading nature of the evaluated ceramic materials, the extraction protocol was strictly normalized by mass. Briefly, the tested materials were incubated in the corresponding culture media at a standardized mass-to-volume ratio of 0.2 g/mL for 24 h at 37 °C. Following incubation, the mixtures were centrifuged at 3000 rpm for 10 min, and the supernatants were subsequently passed through a 0.22 μm filter to sterilize the extracts and remove residual particulates prior to cell exposure. Furthermore, the pH and osmolality of the resulting extraction solutions were routinely monitored to ensure they remained within acceptable physiological ranges prior to all cellular assays (Figure 1).

### 2.7. Cell Proliferation Assay

To evaluate the cytocompatibility and proliferation profiles of the materials, mBMSCs were seeded into 24-well plates at a density of 2 × 10^4^ cells/mL (0.5 mL/well) and cultured in the respective material extraction solutions. To ensure statistical reliability, all cellular assays were performed in independent triplicates (*n* = 3).

Live/Dead Cell Staining Assay: Qualitative cytotoxicity was analyzed via Live/Dead staining. Following a 3-day incubation period, the cells were gently rinsed with PBS and stained with a Calcein AM/PI working solution (C2015S, Beyotime Biotech, Shanghai, China) at 37 °C for 30 min in the dark. Fluorescence imaging was subsequently performed to visually differentiate live (green) and dead (red) cells.

Cell Viability Assay (CCK-8 Assay): Cell viability and proliferation kinetics were quantitatively assessed using the Cell Counting Kit-8 (CCK-8) assay. For this quantitative analysis, wells containing cells cultured in standard medium served as the control group, while cell-free, medium-only wells were utilized as background blanks. Following incubation periods of 1, 3, and 7 days, the culture medium was completely replaced with 500 µL of CCK-8 working solution (C0037, Beyotime Biotech, Shanghai, China) and incubated at 37 °C for 1 h. Subsequently, 100 µL of the reacted supernatant was transferred to a 96-well plate, and the absorbance was measured at 450 nm using a microplate reader. The relative cell viability (%) was calculated using the following equation:

$$Cell\;viability\;\left(\%\right)=\frac{A_{sample}-\;A_{blank}}{A_{control}-\;A_{blank}}\;\times 100\%$$

where *A*_sample_, *A*_control_, and *A*_blank_ represent the absorbance values of the material-treated groups, the control group, and the background blank group, respectively.

### 2.8. In Vitro Angiogenesis Assay

The in vitro angiogenic potential of the materials was evaluated using a HUVEC tube formation assay. Briefly, HUVECs were resuspended in the respective material extraction solutions at a density of 1 × 10^5^ cells/mL and seeded onto Matrigel-coated 48-well plates. Following a 12 h incubation period, the formation of capillary-like networks was observed using an inverted optical microscope (IXplore IX73, Olympus, Tokyo, Japan). To quantify the angiogenic response, representative images were captured from randomly selected fields of view for each well. The relative total tube length (expressed in arbitrary units) and the number of branches per field were subsequently analyzed using ImageJ 1.53 software (National Institutes of Health, Bethesda, MD, USA) (*n* = 3).

### 2.9. Antibacterial Assay

The antibacterial activity against *Staphylococcus aureus* (*S. aureus*, ATCC 25923) and *Escherichia coli* (*E. coli*, ATCC 25922) was evaluated utilizing plate-counting and growth curve kinetic methods. Single colonies were cultured in Luria–Bertani (LB) broth to the logarithmic growth phase, washed, and diluted with sterile PBS to a standardized working concentration of 1 × 10^6^ CFU/mL. The bacterial suspension was co-cultured with each material and incubated at 37 °C under continuous orbital shaking. Bacterial suspensions without any material served as blank controls. To ensure statistical reliability, all antibacterial experiments were performed in independent triplicates (*n* = 3).

Quantitative Plate-Counting Assay: For the quantitative plate-counting assay, the suspensions were subjected to 10-fold serial dilutions utilizing sterile PBS. A 100 µL volume of the appropriately diluted suspension was evenly spread onto solid LB agar plates. Following an 18 h incubation at 37 °C, the plates were photographed, and the visible colony-forming units (CFUs) were manually quantified. Utilizing the quantified CFU data, the antibacterial rate (%) was calculated using the following equation:

$$Antibacterial\;rate\;\left(\%\right)=\frac{{CFU}_{control}-\;{CFU}_{sample}}{{CFU}_{control}}\;\times 100\%$$

where *CFU*_control_ and *CFU*_sample_ represent the number of viable colonies in the control and material-treated groups, respectively.

Growth Curve Kinetic Assay: To dynamically monitor bacterial growth kinetics, samples of the co-culture supernatant were systematically extracted at predefined time intervals over a 14 h period. The optical density (OD) of these samples was immediately measured at 600 nm using a microplate reader to construct high-resolution growth curves.

### 2.10. In Vitro Osteogenic Induction Experiment

To evaluate the osteogenic differentiation capacity of the materials, mBMSCs were seeded into appropriate culture plates (24-well or 6-well) at a standardized density of 2 × 10^4^ cells/mL. Upon reaching appropriate confluence, the basal medium was replaced with the respective material extracts prepared in osteogenic induction medium (MUXMX-90021, OriCell™, Cyagen, Suzhou, China), which were refreshed every two days throughout the induction period. To ensure statistical reliability, all osteogenic differentiation assays were performed in independent triplicates (*n* = 3).

ALP Activity and Alizarin Red Staining Assay: Early osteogenic differentiation was evaluated via alkaline phosphatase (ALP) staining and quantification. Following a 14-day induction period in 24-well plates, the cells were fixed with 4% paraformaldehyde (PFA) and stained using a BCIP/NBT kit (C3206, Beyotime Biotech, Shanghai, China). For quantitative analysis, the cells were lysed with 1% Triton X-100 (Sigma-Aldrich, St. Louis, MO, USA), and intracellular ALP activity was measured utilizing a *p*-nitrophenyl phosphate (pNPP) substrate kit (P0321S, Beyotime Biotech, Shanghai, China) at 405 nm using a microplate reader, normalized to the total protein content. Late-stage mineralization was assessed via Alizarin Red S (ARS) staining. On day 21, the fixed cells were stained with an ARS solution (C0148S, Beyotime Biotech, Shanghai, China) for 30 min. To quantify calcium deposition, the stained nodules were destained using 10% (*w*/*v*) cetylpyridinium chloride (CPC, Beyotime), and the absorbance of the extracted dye was measured at 562 nm using a microplate reader.

Immunofluorescence (IF) Staining Assay: To further assess osteogenic differentiation at the protein level, dual immunofluorescence staining for OCN and RUNX2 was performed. Following the designated induction period, the cells cultured on the respective materials were washed with PBS and fixed with 4% PFA for 30 min. Permeabilization was conducted utilizing 0.5% Triton X-100 (Sigma-Aldrich, St. Louis, MO, USA) for 20 min, followed by blocking with 5% bovine serum albumin (BSA, Solarbio, Beijing, China) for 1 h at room temperature to prevent non-specific binding. Subsequently, the samples were incubated simultaneously with a mixture of primary antibodies consisting of a rabbit anti-RUNX2 monoclonal antibody (ab192256, Abcam, Cambridge, UK; 1:200 dilution) and a goat anti-OCN polyclonal antibody (PA5-47382, Thermo Fisher; Waltham, MA, USA, 1:100 dilution) overnight at 4 °C. Following three washes with PBS, the samples were incubated in the dark with a mixture of secondary antibodies containing Alexa Fluor 488-conjugated donkey anti-rabbit IgG (ab150073, Abcam, Cambridge, UK, 1:1000 dilution) and Alexa Fluor 594-conjugated donkey anti-goat IgG (ab150132, Abcam, Cambridge, UK, 1:1000 dilution) for 1 h at room temperature. The cellular nuclei were counterstained with DAPI (Solarbio, Beijing, China) for 5 min. Finally, fluorescence images were captured utilizing a confocal laser scanning microscope (FV3000, Olympus, Tokyo, Japan). Crucially, to ensure the reliability of subsequent quantitative evaluations, identical optical acquisition parameters (including laser power, exposure time, and gain) were strictly maintained across all experimental groups. RUNX2 expression was visualized as green fluorescence, OCN expression as red fluorescence, and the nuclei were counterstained blue with DAPI. For quantitative analysis, the mean fluorescence intensity (MFI) of RUNX2 and OCN was evaluated utilizing ImageJ. At least three randomly selected representative fields per sample were analyzed, and the fluorescence intensity was normalized to the corresponding number of DAPI-stained nuclei.

Gene Expression Analysis (RT-qPCR): Osteogenic gene expression was quantitatively analyzed utilizing RT-qPCR. Following a 14-day induction period in 6-well plates, total RNA was extracted using TRIzol reagent (TransGen Biotech, Beijing, China). Following vigorous vortexing with chloroform and subsequent centrifugation (12,000× *g*, 15 min, 4 °C), the aqueous phase was collected. The RNA was precipitated with isopropanol, washed with 70% ethanol, and resuspended in RNA dissolving solution. The purified total RNA was reverse-transcribed into complementary DNA (cDNA) using a reverse transcription kit (D7168M, Beyotime Biotech, Shanghai, China) in a thermal cycler. Real-time PCR was executed on an ABI 7500 Real-Time PCR System utilizing a SYBR Green PCR kit (TransGen Biotech, Beijing, China). Relative mRNA (*Bmp2*, *Runx2*, *Col1a1*, Ocn) expression levels were calculated using the 2^−ΔΔCt^ method and normalized to the endogenous *Actb* gene. Primer sequences are detailed in Supplementary Table S1.

### 2.11. Transcriptomic Analysis and Mechanistic Exploration

Transcriptome Sequencing: To explore the underlying molecular mechanisms, transcriptomic analysis was conducted using three independent biological replicates (*n* = 3) per group. After 14 days of osteogenic induction, total RNA was extracted (RIN > 8.0). Sequencing libraries were sequenced on an Illumina NovaSeq 6000 platform, generating > 40 million clean paired-end reads per sample with a >90% mapping rate to the reference mouse genome (GRCm39). Raw reads were filtered, aligned, and quantified. Differentially expressed genes (DEGs) were identified using the DESeq2 R package, applying strict thresholds of *padj* < 0.05 (Benjamini–Hochberg correction) and |log_2_ Fold Change| > 1. All samples were processed simultaneously, negating the need for batch correction. The complete DEG list is provided in Supplementary Materials.

Hypothesis-Driven Mechanistic Exploration via Chemical Modulation: Guided by the transcriptomic cues, a hypothesis-driven approach was utilized to explore whether BG’s enhanced osteogenic effects involved the dual regulation of the NF-κB (suppression) and ECM-integrin (activation) pathways. Specific chemical modulators were introduced: PMA (100 ng/mL, NF-κB agonist) [24] and RGD peptide (500 µg/mL, ECM-integrin competitive inhibitor) [25]. These concentrations were strictly selected based on established dose–response literature to ensure efficient pathway modulation while preventing non-specific cytotoxicity. The mBMSCs (2 × 10^4^ cells/mL) were divided into four groups (*n* = 3):

1. Control group: Standard Osteogenic Medium (OM)
2. BG group: BG extract + OM
3. BG + NF-κB Agonist group: BG extract + OM supplemented with 100 ng/mL Phorbol 12-myristate 13-acetate (PMA; HY-18739, MedChemExpress, Monmouth Junction, NJ, USA)
4. BG + ECM Inhibitor group: BG extract + OM supplemented with 500 µg/mL RGD peptide (HY-P0278, MedChemExpress)

Media were refreshed every two days. Total RNA was extracted on Day 3 to evaluate early cascade genes (*p65*, *Tnf-α*, *Itgb1*, *Fak*) and on Day 14 for downstream osteogenic markers (*Runx2*, *Ocn*). qPCR procedures were identical to Section 2.10, with primer sequences detailed in Supplementary Table S2.

### 2.12. Statistical Analysis

All quantitative data are presented as the mean ± standard error of the mean (sem). Statistical significance was rigorously determined using a one-way analysis of variance (ANOVA) followed by Tukey’s post hoc test for multiple comparisons, utilizing IBM SPSS Statistics 27 software (IBM Corporation, Armonk, NY, USA). All graphical data were plotted using OriginPro 2022b. Statistical significance levels were defined as * *p* < 0.05, ** *p* < 0.01, *** *p* < 0.001.

As this study constitutes pure in vitro basic research without the involvement of human participants or live animal models, specific EQUATOR Network reporting guidelines (e.g., CONSORT, ARRIVE) are not applicable.

## 3. Results and Discussion

### 3.1. Surface Morphology and Phase Analysis

**Figure 2:**
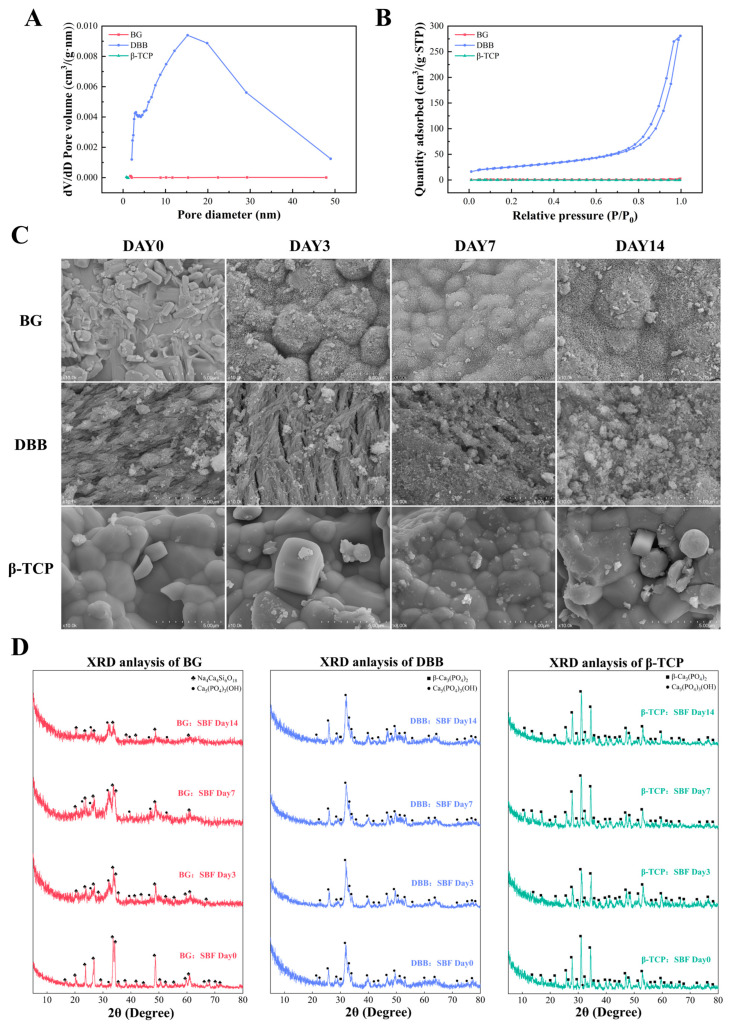
**Physicochemical characterization and structural morphology of the bone graft substitutes.** (**A**) Pore size distributions and (**B**) Nitrogen adsorption–desorption isotherms determined via BET analysis. (**C**) Scanning electron microscopy (SEM) images illustrating the raw surface topography of the commercial substitutes (BG, β-TCP, and DBB). (**D**) X-ray diffraction (XRD) patterns of the unimmersed materials, where the initial crystalline peaks of unimmersed BG correspond to the sodium calcium silicate (Na_4_Ca_4_Si_6_O_18_) phase.

To quantitatively evaluate structural porosity, nitrogen adsorption and desorption analyses were conducted (Figure 2A,B). DBB exhibited a classic Type IV isotherm with a distinct hysteresis loop at higher relative pressures [26], signifying a highly porous architecture with robust surface adsorption capacity. Furthermore, pore size distribution analysis revealed a high density of mesopores in DBB, concentrated predominantly within the 10 to 30 nm range (peaking at approximately 15 nm). This extensive mesoporous network endows DBB with the highest specific surface area among the evaluated materials. In stark contrast, both BG and β-TCP displayed nearly flat adsorption isotherms and lacked detectable mesopores, indicating comparatively dense microstructures devoid of the intrinsic mesoporosity characteristic of DBB.

When immersed in SBF, bioactive materials typically adsorb Ca and P ions to co-precipitate amorphous calcium phosphate (ACP) [27,28], which subsequently transforms into a stable, bone-like hydroxyapatite (HA) layer [29]. Morphological (Figure 2C) and phase composition (Figure 2D) analyses revealed distinct behaviors among the materials. Over the 14-day period, SEM showed that β-TCP maintained its original dense morphology without detectable surface alterations. Conversely, DBB exhibited progressive pore collapse and structural blurring. Crucially, neither material displayed morphological evidence of HA precipitation. This lack of in vitro mineralization was corroborated by their unchanged XRD profiles, which showed no emergence of new crystalline phases from Day 0 to Day 14. Collectively, these results indicate that both DBB and β-TCP lack short-term surface mineralization capabilities in SBF, although the porous structure of DBB is prone to time-dependent physical degradation.

Notably, BG demonstrated substantial surface mineralization during SBF immersion. XRD analysis revealed significant phase transformations, highlighted by the progressive emergence of characteristic HA diffraction peaks between 26° and 32° [30]. Concurrently, SEM observations captured the early nucleation of typical cauliflower-like apatite crystals on the material surface. This rapid generation of a bone-like apatite layer is a fundamental characteristic of bioactive glasses, serving as a classical in vitro indicator of high surface bioactivity, which is widely considered a prerequisite for favorable cellular interactions and subsequent osteogenic potential [31,32].

**Figure 3:**
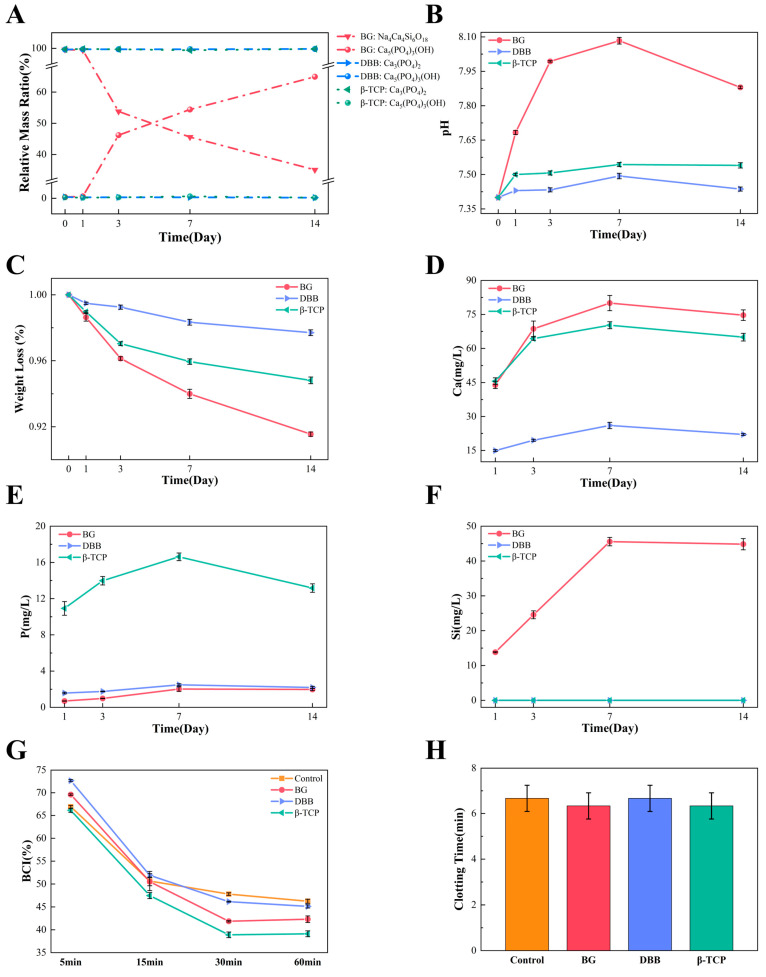
**In vitro degradation behavior, dynamic phase transformation, and hemocompatibility.** (**A**) Relative quantitative XRD phase analysis displaying the dynamic mass fraction transition from the initial crystalline phase to the newly precipitated hydroxyapatite (HA) phase on BG over time. (**B**) Local microenvironmental pH variations. (**C**) Remaining mass fraction profiles of the substitutes during the 14-day immersion period, demonstrating the intrinsic chemical stability of DBB despite its extensive porosity. Cumulative release kinetics into the extraction medium for (**D**) Calcium (Ca), (**E**) Phosphorus (P), and (**F**) Silicon (Si) ions (Si was undetected in the DBB and β-TCP groups, resulting in overlapping zero-baselines). (**G**) Quantitative evaluation of the Blood Clotting Index (BCI) and (**H**) comprehensive blood coagulation time measurements to assess initial hemocompatibility. Data are presented as mean ± sem (*n* = 3).

These morphological and structural observations were further supported by relative quantitative XRD phase analysis (Figure 3A). For BG, the dynamic phase transition was striking: the relative mass fraction of the newly precipitated HA phase (Ca_5_(PO_4_)_3_(OH)) increased continuously, mirrored by a concomitant decrease in the initial sodium calcium silicate phase (Na_4_Ca_4_Si_6_O_18_). Regarding this initial crystalline phase, it is important to note that while raw 45S5 bioactive glass powders are typically fully amorphous, the commercial Perioglas^®^ utilized in this study exhibits distinct crystalline peaks prior to immersion. This is attributed to the high-temperature sintering and granulation processes required to manufacture these clinical-grade bone grafts, which inevitably induce partial devitrification of the amorphous glass network and the subsequent precipitation of crystalline sodium calcium silicate phases [11]. These results directly substantiate the prominent mineralization observed via SEM, thereby confirming the pronounced surface bioactivity of the BG [33]. Conversely, DBB and β-TCP displayed steady compositional stability, maintaining their phase equilibrium over time, with no detectable accumulation of secondary HA. These stable relative ratios further validate the absence of short-term surface phase transformations in DBB and β-TCP. Taken together, these in vitro evaluations highlight fundamentally distinct surface behaviors: DBB provides a naturally porous but chemically stable matrix, β-TCP remains structurally dense and phase-stable, whereas BG is uniquely distinguished by its highly reactive surface and rapid HA mineralization capacity.

### 3.2. In Vitro Degradation

In vitro degradation kinetics provide fundamental insights into the physicochemical stability and ion release behaviors of bone graft substitutes. Over the 14-day evaluation period, the three materials exhibited distinct dissolution profiles (Figure 3B–F). DBB displayed high chemical inertness under these aqueous conditions [34], characterized by negligible mass loss (Figure 3C), a constant pH comparable to the initial buffer (Figure 3B), and minimal Ca and P release, with no detectable Si leaching (Figure 3D–F). While this high structural durability provides a stable osteoconductive matrix, the previous literature notes that the persistence of undegraded residual material may eventually influence the rate of native bone replacement [9,35,36]. In contrast, β-TCP presented a moderate, time-dependent degradation profile, demonstrating steady weight loss accompanied by sustained P release, moderate Ca leaching, and similarly undetectable Si release, alongside a mild elevation in local pH. Conversely, BG exhibited rapid dissolution kinetics. It experienced rapid material mass loss driven by a substantial, continuous burst of Ca and unique Si ions (reaching approximately 45 mg/L by day 7), which triggered a sharp initial spike in microenvironmental pH to roughly 8.1. While this rapid release of functional ions is closely associated with the high in vitro bioactivity of BG [37], such an accelerated degradation rate raises potential limitations regarding its long-term volumetric stability [7,19]. Interestingly, our previous BET analysis revealed that while BG and β-TCP share comparable dense microstructures, DBB possesses the highest specific surface area and an extensive mesoporous network. Despite this structural advantage, which theoretically accelerates dissolution, DBB still exhibited the slowest degradation rate compared to BG and β-TCP. This structural-kinetic mismatch highlights that the fundamentally divergent dissolution profiles observed among the groups are intrinsically governed by their distinct chemical compositions rather than their physical porosity. Overall, these in vitro dissolution kinetics reveal a critical bioactivity-stability trade-off among the three material classes: DBB demonstrates prominent chemical durability, β-TCP offers a moderate degradation rate, while BG exhibits significant ion release at the expense of accelerated mass loss.

### 3.3. In Vitro Coagulation Performance

The preliminary in vitro hemocompatibility of the materials was evaluated through an in vitro dynamic BCI assay and macroscopic clotting time measurements. As illustrated in Figure 3G, the BCI values of all groups decreased progressively over time, reflecting normal and continuous clot formation. However, quantitative analysis of the absolute clotting time (Figure 3H) revealed that all groups achieved stable clot formation within a tight physiological window, demonstrating that these minor kinetic variations were not statistically significant (*p* > 0.05). The lack of significant deviation from the physiological control indicates that none of these materials induce notable procoagulant or anticoagulant effects, supporting their basic compatibility with blood elements in vitro.

### 3.4. Cell Viability

**Figure 4:**
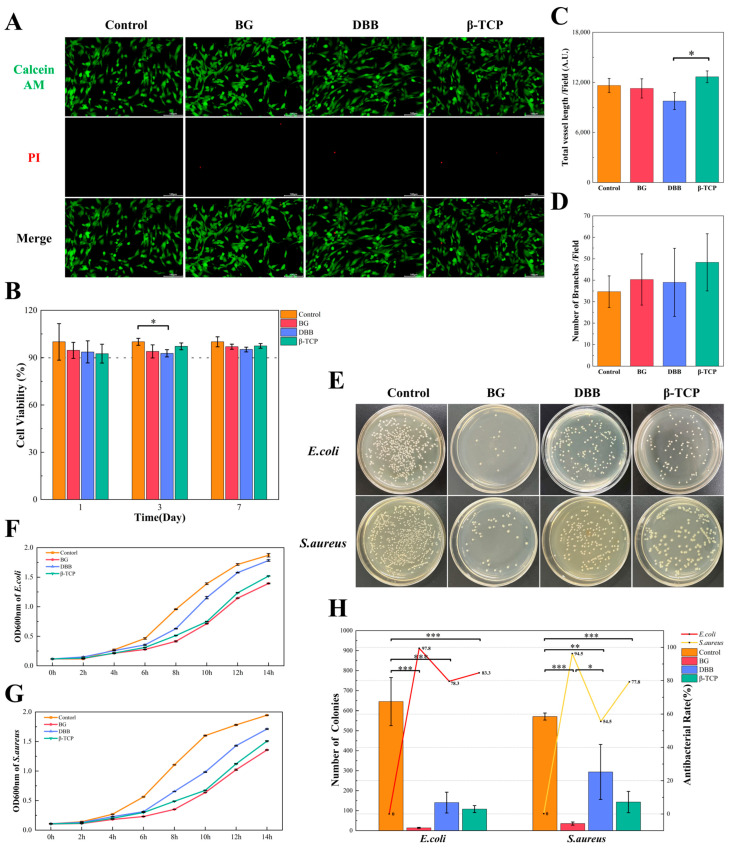
**In vitro cytocompatibility, angiogenic potential, and antibacterial efficacy.** (**A**) Live/Dead fluorescence staining of mBMSCs (green: live; red: dead), showing high cell viability and healthy morphology across all groups. (**B**) Cell viability evaluated via CCK-8 assay over 7 days, demonstrating sustained cellular proliferation. (**C**,**D**) Quantitative evaluation from the HUVEC tube formation assay detailing (**C**) relative total tube length (expressed in arbitrary units, A.U.) and (**D**) number of branched nodes per randomly selected field. The results indicate a satisfactory baseline angiogenic potential for all materials, with β-TCP showing a significant enhancement in tube length relative to DBB. (**E**) Representative agar plate photographs for *E. coli* and *S. aureus* after treatment with material extracts. (**F**,**G**) Dynamic bacterial growth kinetics monitored via optical density (OD_600nm_) over a 14 h incubation period for (**F**) *E. coli* and (**G**) *S. aureus*, corroborating the strong bacterial suppression by BG. (**H**) Quantitative colony counts and antibacterial rates. BG exhibits superior bactericidal efficacy (>90%) compared to the weaker inhibition by DBB and β-TCP. Data are presented as mean ± SEM (*n* = 3 independent replicates). Data are presented as mean ± sem (*n* = 3). * *p* < 0.05, ** *p* < 0.01, *** *p* < 0.001.

To systematically evaluate the in vitro cytocompatibility of the materials, Live/Dead fluorescence staining and CCK-8 assays were conducted using mBMSCs (Figure 4A,B). As visually depicted in Figure 4A, the Live/Dead staining revealed no distinct qualitative differences among the groups. Across all tested materials and the blank control, the mBMSCs exhibited healthy, spindle-shaped morphologies with an overwhelming predominance of viable cells (intense green fluorescence) and negligible cell death (rare red fluorescence). This favorable cytocompatibility was robustly corroborated by the quantitative CCK-8 assay (Figure 4B), which demonstrated that the relative cell viability for all experimental groups consistently remained above 90% throughout the 7-day culture period. Collectively, these results demonstrate that all tested materials exhibit favorable cytocompatibility, consistent with findings from previous studies [38,39,40], and effectively support cellular proliferation.

Notably, the DBB group exhibited a transient, statistically significant decrease in cell viability on Day 3 relative to the control group (*p* < 0.05). However, this metabolic dip was fully resolved by Day 7, indicating a robust cellular recovery. Since this biological evaluation was conducted using material extracts, this temporary deceleration in proliferation is likely attributed to the unique physical topography of DBB during the extract preparation phase. As confirmed by our previous BET analysis, the inherent highly porous architecture and extensive mesoporous network of DBB grant it an exceptionally high specific surface area. During the initial immersion for extraction, this surface can competitively adsorb a substantial amount of essential nutrients, serum proteins, and growth factors from the culture medium. Consequently, the resulting DBB extract may present a transiently depleted nutritional microenvironment, temporarily limiting early cellular proliferation [41]. Ultimately, the rapid recovery and sustained growth by Day 7 confirm that the extracts of all evaluated materials, including the highly porous xenograft DBB, possess satisfactory cytocompatibility and effectively support the continuous proliferation of mBMSCs.

### 3.5. In Vitro Angiogenic Potential

Furthermore, successful bone regeneration is critically dependent on coupling with adequate early angiogenesis. To evaluate this, an in vitro HUVEC tube formation assay was employed. As illustrated in Figure 4C,D, the overall angiogenic capacity was largely maintained across the experimental groups, although specific quantitative variations were observed. Regarding the complexity of the vascular networks, the number of branches per field (Figure 4D) showed no statistically significant differences between the control and experimental groups. However, quantitative analysis of the overall tube networks (Figure 4D) revealed that the β-TCP group possessed a significantly enhanced tube formation capability compared to the DBB group (*p* < 0.05). Meanwhile, the BG group exhibited intermediate angiogenic performance, remaining highly comparable to the blank control. Collectively, these findings indicate that while β-TCP exerts a mild stimulatory effect on endothelial tube elongation [42] relative to the natural DBB scaffold, all three bone graft substitutes safely maintain a satisfactory baseline potential for supporting early vascular network formation without inducing inhibitory effects.

### 3.6. Antibacterial Activity

In addition to fundamental cytocompatibility, effective infection prevention is a critical prerequisite for the clinical success of bone grafting procedures. The baseline antibacterial performance against the representative Gram-negative *E. coli* and Gram-positive *S. aureus* was systematically evaluated. As is visually evident in the plate counting assays (Figure 4E), the Control group displayed vigorous bacterial proliferation, whereas the BG group exhibited significant bactericidal activity, substantially reducing the colonies of both strains. Quantitative analysis (Figure 4H) confirmed these observations: BG achieved high antibacterial rates of 97.8% against *E. coli* and 94.5% against *S. aureus*, significantly outperforming all other groups (*p* < 0.001). β-TCP demonstrated moderate, broad-spectrum antibacterial efficacy (83.3% and 77.8%, respectively). Conversely, DBB exhibited the weakest inhibition, particularly against *S. aureus* (54.5%). These static endpoints were further validated by dynamic bacterial growth kinetics, monitored via optical density (OD_600nm_) over a 14 h incubation period (Figure 4F,G). Consistent with the plate counts, the BG group most effectively suppressed bacterial proliferation. It notably delayed the onset of the exponential logarithmic growth phase and significantly restricted the maximum peak turbidity for both strains compared to the Control. The DBB and β-TCP groups displayed intermediate kinetic profiles, exerting a weaker, primarily bacteriostatic effect.

The significant antibacterial efficacy of BG is primarily governed by a multifaceted mechanism driven by its highly accelerated dissolution kinetics. First, the rapid burst release of alkaline ions (predominantly Na^+^ and Ca^2+^) sharply elevates the local microenvironmental pH (reaching approximately 8.1, Figure 3B), creating an alkaline stress that disrupts bacterial transmembrane electrochemical gradients [43,44]. Second, this intense initial ion leaching induces severe local hyperosmotic shock, which physically compromises bacterial membrane integrity [45]. Importantly, recent advanced investigations have elucidated that, beyond basic pH and osmolarity shifts, the specific release and accumulation of soluble Si species may contribute significantly to bacterial inhibition [46]. The substantial deposition of Si directly onto bacterial cell surfaces physically punctures the cell envelope, leading to severe cell wall degradation and irreversible cytoplasmic leakage [46,47]. Concurrently, this pronounced ionic imbalance triggers the rapid delocalization of vital marker proteins, effectively paralyzing central bacterial metabolism, redox homeostasis, and translational stability [46]. Together, this synergistic integration of osmotic-alkaline stress, metabolic impairment, and physical structural disruption provides a reasonable explanation for the significant bactericidal activity observed in the BG group.

Although the significant inhibition of representative Gram-positive (*S. aureus*) and Gram-negative (*E. coli*) models in this study sufficiently demonstrates the fundamental antibacterial efficacy of the materials, given that these substitutes are primarily designed for dental and maxillofacial applications, specific qualitative and quantitative evaluations on oral pathogens (e.g., *P. gingivalis*, *F. nucleatum*, and *T. denticola*) should be systematically integrated in future investigations.

### 3.7. In Vitro Osteogenic Differentiation and Molecular Validation

**Figure 5:**
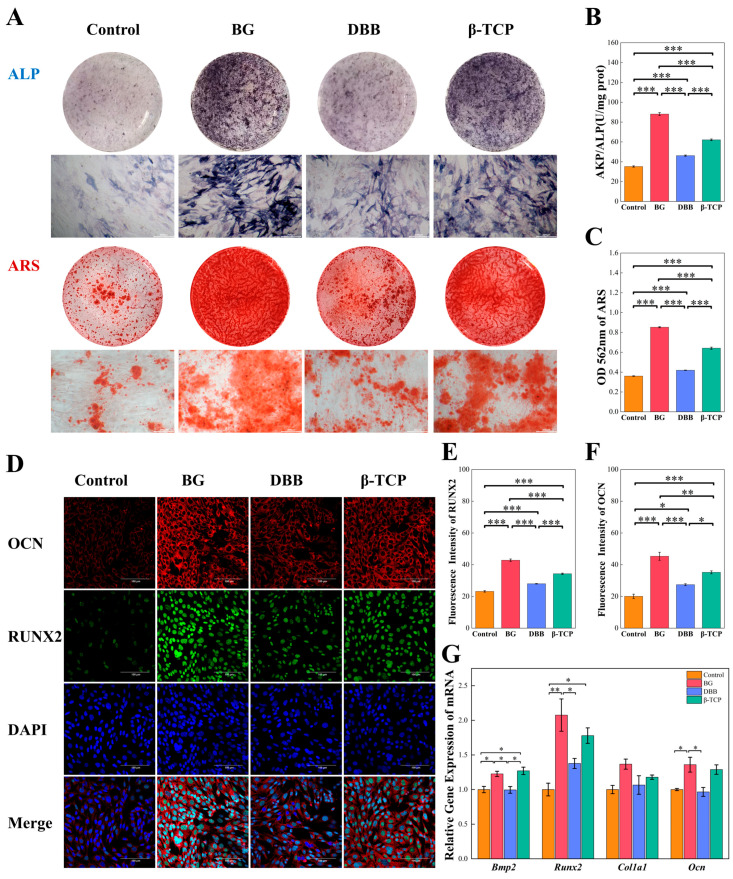
**In vitro osteogenic differentiation and molecular validation.** (**A**) Representative images of ALP staining (day 14) and ARS staining (day 21). BG exhibits the most abundant early-stage ALP expression and late-stage calcium nodule deposition. (**B**,**C**) Quantitative analysis of (**B**) ALP enzymatic activity and (**C**) ARS calcium deposition. The results confirm significantly enhanced osteogenic induction by BG and moderate enhancement by β-TCP, compared to the baseline levels of DBB. (**D**) Representative confocal immunofluorescence images of OCN (red) and Runx2 (green) expression. Nuclei were counterstained with DAPI (blue). (**E**,**F**) Quantitative fluorescence intensity analysis of (**E**) Ocn and (**F**) Runx2, demonstrating significantly upregulated protein-level expression in the BG group. (**G**) Relative mRNA expression of key osteogenic marker genes (*Bmp2*, *Runx2*, *Col1a1*, and *Ocn*) evaluated via qPCR after 14 days of induction. BG significantly upregulates transcriptional expression, robustly corroborating the phenotypic mineralization results. All quantitative data are expressed as mean ± sem (*n* = 3). * *p* < 0.05, ** *p* < 0.01, *** *p* < 0.001.

The ultimate clinical goal of a bone graft substitute is to support new bone formation. We systematically evaluated the osteoinductive potential of the materials to enhance osteogenic differentiation, starting with phenotypic assessments of early-stage ALP activity and late-stage calcium nodule deposition. ALP is a critical enzyme in the initial mineralization phase of the bone extracellular matrix [48]. As demonstrated in Figure 5A,B, day 14 ALP staining and quantitative activity analysis revealed that BG induced the highest density of ALP-positive nodules and the strongest enzymatic activity, followed by β-TCP. DBB, conversely, exhibited a baseline pattern comparable to the Control. Consistent with these early-stage markers, late-stage ARS staining and corresponding quantification on day 21 (Figure 5A,C) revealed abundant, densely packed calcium nodules predominantly on the BG surface, reflecting highly active late-stage mineralization [49]. While β-TCP induced moderate calcium deposition, DBB showed minimal enhancement over the Control. These phenotypic results indicate that while DBB provides a favorable osteoconductive framework, it fundamentally lacks the dynamic ability to promote osteogenic differentiation inherent to rapidly degrading, ion-releasing materials like BG [50,51].

To bridge the gap between macroscopic mineralization and gene transcription, we further evaluated the protein-level expression of core osteogenic markers via dual immunofluorescence staining (Figure 5D). RUNX2 serves as an essential early transcription factor, while OCN (osteocalcin) is a definitive marker of late-stage osteoblast maturation [52]. Confocal microscopy revealed that the BG group exhibited the most intense intracellular fluorescence for both RUNX2 (green) and OCN (red) compared to the other groups. Quantitative fluorescence intensity analysis (Figure 5E,F) further supported these visual observations, demonstrating that BG significantly upregulated the protein expression of both RUNX2 and OCN (*p* < 0.001) relative to the Control and DBB groups. β-TCP also displayed a moderate, yet significant, enhancement in protein expression, whereas DBB showed no obvious immunofluorescence enhancement over the baseline Control.

To validate these macroscopic observations at the molecular level, the expression of key osteogenic marker genes was quantified via qPCR (Figure 5G). *Bmp2* and *Runx2* are core transcriptional regulators orchestrating the osteogenic differentiation cascade [53]. After 14 days of induction, the expression of *Bmp2* was significantly upregulated in both the BG and β-TCP groups relative to the Control (*p* < 0.05). Furthermore, *Runx2* expression was markedly elevated in the BG group (*p* < 0.01) and moderately upregulated in the β-TCP group, whereas DBB elicited no significant difference. As a definitive late-stage mineralization marker, *Ocn* was also significantly elevated in the BG group (*p* < 0.05), but remained at baseline levels in the DBB and β-TCP groups [54]. Interestingly, no statistically significant differences were observed in *Col1a1* expression among the experimental groups at this time point. The substantial transcriptional upregulation of *Bmp2*, *Runx2*, and *Ocn* in the BG group closely corroborates with the staining results. This prominent osteogenic enhancement strongly correlates with its rapid in vitro degradation kinetics (Figure 3D,F), suggesting that the continuous burst release of Ca and Si ions from BG serves as a significant biochemical stimulus for osteogenic gene activation [6].

### 3.8. Transcriptomic Profiling of Osteogenesis

**Figure 6:**
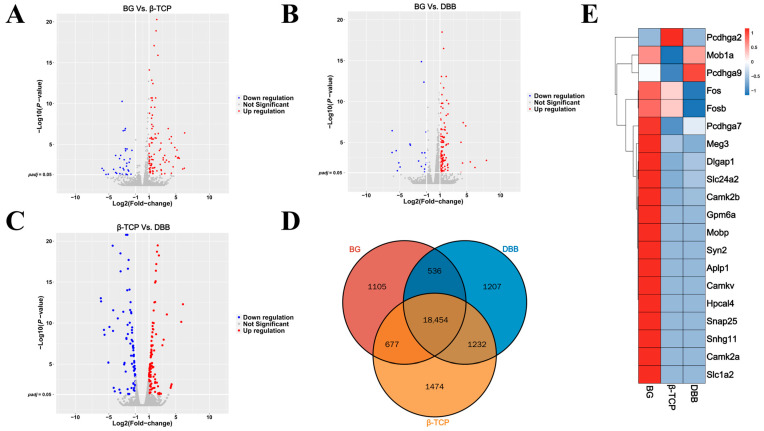
**Transcriptomic profiling of mBMSCs cultured on different materials.** (**A**–**C**) Volcano plots illustrating differentially expressed genes (DEGs) between the pairwise comparison groups (BG vs. β-TCP, BG vs. DBB, and β-TCP vs. DBB) across three independent biological replicates (*n* = 3). The screening thresholds were strictly set at a |log_2_ fold change| > 1 and a Benjamini–Hochberg adjusted *p*-value (*padj*) < 0.05. (**D**) Venn diagram demonstrating the overlap of DEGs among the experimental groups. The 1105 BG-specific expressed genes were identified via a coe Venn test using a baseline expression threshold > 1. (**E**) Heatmap of representative DEGs, highlighting the distinct genetic expression profile of cells cultured on BG.

To further untangle the global molecular mechanisms potentially associated with the enhanced osteogenic differentiation, genome-wide transcriptomic analysis (RNA-seq) was performed to comprehensively map the transcriptomic profiles of mBMSCs treated with the respective material extraction solutions. Volcano plots (Figure 6A–C) visualized the substantial global transcriptional shifts induced by BG compared to the relatively inert DBB and intermediate β-TCP materials. A Venn diagram (Figure 6D) highlighted a distinct subset of 1105 uniquely expressed genes in the BG group, underscoring that BG activates highly specific genetic cascades not triggered by the structural cues of DBB or β-TCP alone. Furthermore, a hierarchical clustering heatmap (Figure 6E) distinguished the BG group against the others, demonstrating a distinct, clustered upregulation of specific pro-differentiation genes (such as Fos and Fosb).

**Figure 7:**
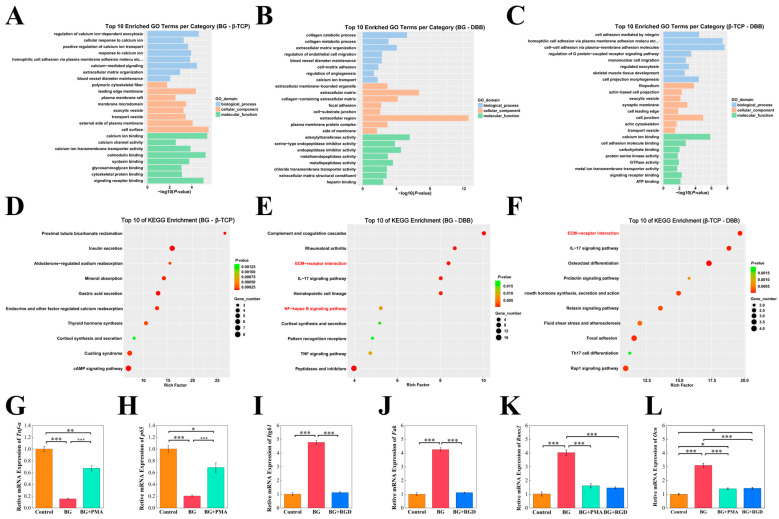
**Transcriptomic enrichment and hypothesis-driven mechanistic exploration.** (**A**–**C**) Top 10 Enriched GO Terms and (**D**–**F**) KEGG Enrichment analysis for the sequential pairwise comparisons of BG vs. β-TCP (**A**,**D**), BG vs. DBB (**B**,**E**), and β-TCP vs. DBB (**C**,**F**), The enriched terms and pathways are ranked by significance from highest to lowest based on the Benjamini–Hochberg adjusted *p*-value (*padj* < 0.05), highlighting the critical involvement of ECM-receptor interactions and the NF-κB signaling pathway. (**G**,**H**) Relative mRNA expression of inflammation markers *Tnf-α* and *p65*. (**I**,**J**) Relative mRNA expression of adhesion mediators *Itgb1* and *Fak*. (**K**,**L**) Relative mRNA expression of *Runx2* and *Ocn* following treatment with an NF-κB agonist (PMA) or an integrin inhibitor (RGD peptide), exploring the potential the dual regulatory mechanism of BG. Data are presented as mean ± sem (*n* = 3). * *p* < 0.05, ** *p* < 0.01, *** *p* < 0.001.

Subsequent Gene Ontology (GO) and Kyoto Encyclopedia of Genes and Genomes (KEGG) enrichment analyses (Figure 7A–F) were conducted to explore the functional pathways associated with these transcriptomic variations. Notably, while unbiased enrichment analyses yielded multiple enriched biological processes, we specifically focused our downstream exploration on the NF-κB signaling pathway and ECM-receptor interactions based on these transcriptomic cues. This hypothesis-driven selection suggested that the enhanced osteogenic performance of BG might be correlated with the regulation of these specific cascades. This correlation presents a plausible hypothesis that BG’s osteogenic activity may be associated with a potential dual mechanism involving both immunomodulatory and mechanotransductive signaling. However, it must be emphasized that these transcriptomic and initial transcription-level assessments are primarily hypothesis-generating, and further rigorous molecular validations are required to establish definitive causality.

### 3.9. Exploration of Osteogenic Mechanisms: Immunomodulation and Mechanotransduction

The enhanced osteogenic performance of BG cannot be solely explained by its static surface topography; rather, it is likely dynamically driven by its accelerated degradation and the resulting unique ionic microenvironment. Subsequent exploration through qPCR and chemical modulation further supported the hypothesis regarding the potential involvement of the enriched pathways identified via RNA-seq.

Biologically, the NF-κB pathway serves as a classical mediator of inflammatory responses, and its chronic activation is known to severely impair osteogenic differentiation [55]. Our experimental findings revealed that BG treatment significantly downregulated the expression of key pro-inflammatory pathway components, specifically *Tnf-α* and the *p65* subunit of NF-κB (Figure 7G,H). This suggests that the ionic dissolution products of BG may actively suppress inflammatory signaling, creating a “pro-osteogenic” low-inflammatory microenvironment. Specifically, the substantial release of Si ions from bioactive glasses has been widely documented to exert significant immunomodulatory effects, facilitating tissue regeneration by downregulating NF-κB-mediated inflammatory cascades [56,57]. Importantly, when this pathway was reactivated utilizing PMA, the osteogenic advantages conferred by BG were effectively negated, evidenced by significantly reduced expression of *Runx2* and *Ocn* (Figure 7K,L). This supports the hypothesis that the immunomodulatory capacity of BG, particularly its suppression of NF-κB, might be a crucial biological factor for its enhancement of osteogenic differentiation.

Simultaneously, the physical interaction between the dynamic material surface and cellular components also plays an essential role. Concurrently with Si ion release, the rapid degradation of BG continuously supplies Ca ions and drives the rapid precipitation of a bioactive HA layer (as observed in Figure 2C,D). This dynamic, calcium-rich mineralized surface is favorable for the adsorption of extracellular matrix proteins (e.g., fibronectin), which subsequently triggers integrin clustering and focal adhesion maturation [58,59]. Our investigation demonstrated that BG significantly upregulates the expression of *Itgb1* and its downstream signaling mediator, Focal Adhesion Kinase (*Fak*) (Figure 7I,J).

Furthermore, when RGD peptides were utilized to competitively block integrin binding sites, this mechanotransduction interaction was effectively impeded, resulting in a significant reduction in the expression of key osteogenic markers (Figure 7K,L).

Collectively, these integrated results support the hypothesis that BG enhances osteogenic differentiation not merely through basic osteoconduction, but is potentially driven by molecular mechanisms explored in this study, specifically: (1) biologically suppressing NF-κB-mediated inflammation, and (2) enhancing ECM-integrin mechanotransduction.

## 4. Conclusions

In summary, while BG, β-TCP, and DBB all exhibited favorable short-term in vitro cytocompatibility, they demonstrated distinct biological profiles. Notably, BG demonstrated significant potential to enhance osteogenic differentiation and substantial antibacterial performance against the tested strains, suggesting a potential working model involving the simultaneous suppression of NF-κB-mediated inflammation and activation of the ECM-Integrin-FAK axis. However, the accelerated degradation of BG presents a potential limitation regarding long-term structural maintenance in vitro, which contrasts with the structural durability of DBB. Furthermore, it is important to acknowledge that these mechanistic insights are derived from an in vitro murine cellular model, which inherently lacks the full physiological complexity of human in vivo bone healing. Ultimately, resolving this bioactivity-stability trade-off, potentially coupled with rigorous in vivo validations, remains critical for rationally designing next-generation bone scaffolds to facilitate future translational investigations.

## Data Availability

The raw data supporting the conclusions of this article will be made available by the authors on request.

## Supplementary Materials

The following supporting information can be downloaded at: https://www.mdpi.com/article/10.3390/jfb17070312/s1, Table S1: Primer sequences used for quantitative real-time PCR (RT-qPCR) of osteogenesis-related genes; Table S2: Primer sequences used for RT-qPCR validation of pathway-related genes identified via transcriptome sequencing; File S1: RNA quality control (QC) reports for transcriptome sequencing; Table S3: Gene Ontology (GO) enrichment analysis data of the differentially expressed genes (DEGs); Table S4: Kyoto Encyclopedia of Genes and Genomes (KEGG) pathway enrichment analysis data of the differentially expressed genes (DEGs).
