# Supplementary material for: Comparative In Vitro Evaluation and Osteogenic Mechanisms of Representative Bone Graft Substitutes: Bioactive Glass, Beta-Tricalcium Phosphate, and Deproteinized Bovine Bone
Source: J Funct Biomater. 2026 Jun 26;17(7):312. doi: 10.3390/jfb17070312 (PMC13412606; doi:10.3390/jfb17070312)
Supplement: Supplementary file 1 [file jfb-17-00312-s001.zip › Table S1.pdf]

Table S1. Primer sequences used for the qPCR analysis of key osteogenic marker genes.

Gene	The primer sequences	
Actb	F	TCAACACCCCAGCCATGTAC
	R	AATGCCTGGGTACATGGTGG
Runx2	F	TGGTAAAGGCTCAGGCATGG
	R	AACAGAGAGCGAGGGGGTAT
Bmp2	F	GAGAAGCTAGAGTCGCGGAC
	R	AGAAGTCTCCAGCCAAGTGC
Col1a1	F	GTCTGCCCAGCAAACAAAGG
	R	TGCTGGTCTAGGGAGCATCT
Ocn	F	AGACAAGTCCCACACAGCAG
	R	AGGGCAGAGAGAGAGGACAG
