# Supplementary material for: Comparative In Vitro Evaluation and Osteogenic Mechanisms of Representative Bone Graft Substitutes: Bioactive Glass, Beta-Tricalcium Phosphate, and Deproteinized Bovine Bone
Source: J Funct Biomater. 2026 Jun 26;17(7):312. doi: 10.3390/jfb17070312 (PMC13412606; doi:10.3390/jfb17070312)
Supplement: Supplementary file 1 [file jfb-17-00312-s001.zip › Table S2.pdf]

Table S2. Primer sequences used for the qPCR validation of molecular regulatory pathways.

Gene	The primer sequences	
Actb	F	TCAACACCCCAGCCATGTAC
	R	AATGCCTGGGTACATGGTGG
p65	F	AGGCTTCTGGGCCTTATGTG
	R	TGCTTCTCTCGCCAGGAATAC
Tnf-α	F	CCCTCACACTCAGATCATCTTCT
	R	GCTACGACGTGGGCTACAG
Itgb1	F	ATGCCAAATCTTGCGGAGAAT
	R	TTTGCTGCGATTGGTGACATT
Fak	F	GTCCTGGAGGAGTTTTCCTCT
	R	GCACTTCTTTCAACACGTCTTC
