# Supplementary material for: Comparative In Vitro Evaluation and Osteogenic Mechanisms of Representative Bone Graft Substitutes: Bioactive Glass, Beta-Tricalcium Phosphate, and Deproteinized Bovine Bone
Source: J Funct Biomater. 2026 Jun 26;17(7):312. doi: 10.3390/jfb17070312 (PMC13412606; doi:10.3390/jfb17070312)
Supplement: Supplementary file 1 [file jfb-17-00312-s001.zip › File S1.zip!/DDB2_no_rRNA.1qcQC_report.html]

DDB2\_no\_rRNA.1.fq.gz FastQC Report


## Summary

- Basic Statistics
- Per base sequence quality
- Per sequence quality scores
- Per base sequence content
- Sequence Length Distribution

## Basic Statistics

| Measure | Value |
| --- | --- |
| Filename | DDB2\_no\_rRNA.1.fq.gz |
| File type | Conventional base calls |
| Encoding | Sanger / Illumina 1.9 |
| Total Sequences | 21032895 |
| Filtered Sequences | 0 |
| Sequence length | 25-150 |
| %GC | 49 |

## Per base sequence quality

## Per sequence quality scores

## Per base sequence content

## Sequence Length Distribution
